# Poly (aniline-co-aniline-2,5-disulfonic acid) / L-ascorbic acid / Ag@SiO_2_ / polysafranin nanocomposite: synthesis, characterization and anomalous electrical behaviour

**DOI:** 10.1186/s13065-024-01174-7

**Published:** 2024-04-20

**Authors:** Hammed H. A. M. Hassan, Marwa Abdel Fattah, Fatma Abdel Maged

**Affiliations:** 1 Chemistry department, Faculty of Science, Alexandria University, P.O. 2, Moharram Beck, Alexandria, 21568 Egypt; 2 Menoufia Higher Institute of Engineering and Technology MNF-HIET, Menoufia, Egypt; 3 Canal High Institute of Engineering and Technology, Suez, 43713 Egypt

**Keywords:** Electrical conductivity, Nanocomposite, Permittivity, Safranin, Self-doping

## Abstract

We report the synthesis of sulfonated copolyaniline/polysafranin/L-ascorbic acid/Ag@SiO_2_ fine powdered nanocomposites and investigate the influence of incorporating the dye on their conductivity. The composite was characterized via IR, UV, cyclic voltammetry (CV), electric, dielectric, SEM, TEM, TGA and DSC measurements. Microscopy images revealed intensified spherical particles that were dispersed across the entire surface, and the SiO_2_/Ag particles were distributed on the surface. The XRD results exhibited peaks at many 2q values, and their interatomic spacing (d) and crystallite (grain) sizes were calculated. The thermal degradation curves exhibited an interesting model of stability. The cyclic voltammogram exhibited redox peaks identical to those of the reported analogues. The d.c. conductivity of the oligomer varied from 0.06 − 0.016 (s/cm), and that of the composite varied from 0.008 to 0.016 (s/cm). The material changed from a semiconductor to a metallic material. The observed conductivity is mainly attributed to self-doping between the sulfonate groups and the charged nitrogen atoms in the polymer chains. The frequency dependence of the permittivity, ε′, showed a marked effect on the frequency window under consideration. The permittivity, ε′, is independent of the increase in the frequency of the oligomer and the composite. This behavior supports the non-Debye dependency by confirming the occurrence of electrode polarization and space charge effects. In conclusion, the incorporation of safranin dye with a thermally stable, highly sulfonated polyaniline derivative/Ag@SO_2_ nanocomposite achieved improved conductivity after heating. The d.c. conductivities are comparable to those of many commercial inorganic or organic composites, and because of their attractive electrical properties, we suggest that these materials are promising for electronic field applications.

## Introduction

Due to its simple and cost-effective commercial production, polyaniline (PANI) and its derivatives are an important class of conducting polymers, and their interesting properties have made them applicable in multiple fields, such as rechargeable batteries, supercapacitors, solar cells, analytical sciences, biomedicine, conductive paints and adhesives, and environmental issues such as wastewater treatment and many other applications [1–3]. Tuning of their properties, such as by controlling their morphology and enhancing their conductivity, is desirable research target. Mixing such polymers with organic and/or inorganic materials has produced composites and blends that have unique properties and applications [4]. The conductivity of PANI ranges from σ ≤ 10^− 10^ S cm^− 1^ (undoped base form) to σ ≥ 10 S cm^− 1^ (acidic doped salt form) [5]. Although acidic doping improves the polymer characteristics, it limits the heat resistance and electrical conductivity [6]. One additional problem is that acid dopants are potentially corrosive, and their use raises a risk to the environment. Self-doped polyanilines are polyaniline derivatives that bear negatively charged functional groups. The polymerization of aniline derivatives containing sulfonic (-SO_3_H) groups leads to self-doped PANI. In contrast to PANI, its self-doped derivatives contain an ionizable, negatively charged functional group, which acts as an inner dopant anion bound to the polymer backbone. No anion exchange between the polymer and its surroundings occurs during oxidation or reduction. Charge compensation occurs at the expense of cation (usually a proton) exchange, which occurs much faster than the other processes and does not limit the rate of the charging (redox) process [7]. Self-doped conducting PANIs with acid moieties are considered rational alternatives because they are soluble or dispersed in organic solvents and hence can expand the utility of self-doped PANI. However, self-doped conducting polyanilines, which are soluble in organic solvents, have been little studied thus far [8]. Due to the influence of electronic and steric hinderance of the -SO_3_H group, the direct synthesis of self-doped PANI has failed; however, the copolymerization of self-doped polyaniline with aniline based on the autocatalytic polymerization reaction [9] has been considered a solution. Direct polymerization of aminobenzenesulfonic acid was neither chemically nor electrochemically successful for two reasons: (i) The strong electron-withdrawing properties of the -S0_3_H decreased the electron density on the amino groups, and the monomers could not be oxidized. (ii) The presence of the bulky group of -S0_3_H on the phenyl rings affects the reactivity of two cationic radical monomers for head-tail coupling to form a relatively stable intermediate complex, which likely limits the polymerization process and results in a low-molecular weight polymer. PANI itself can be synthesized by chemical oxidation polymerization of aniline via oxidant radical initiators. Therefore, once the polymerization of polyaniline starts, an autocatalytic reaction takes place, and therefore, copolymerization of aniline and aminobenzenesulfonic acid can be achieved. Nevertheless, due to the influential nature of the sulfonic group, such copolymerization results in a low-molecular-weight polymer with a conductivity of only 10^− 4^ S cm^− 1^ [10]. The lower conductivity of the self-doped polymer is attributed to the decreased interchain diffusion of the charge carriers due to the presence of side groups, which force the chain out of planarity and result in lower crystallographic order between the chains [10].

**Fig. 1:**
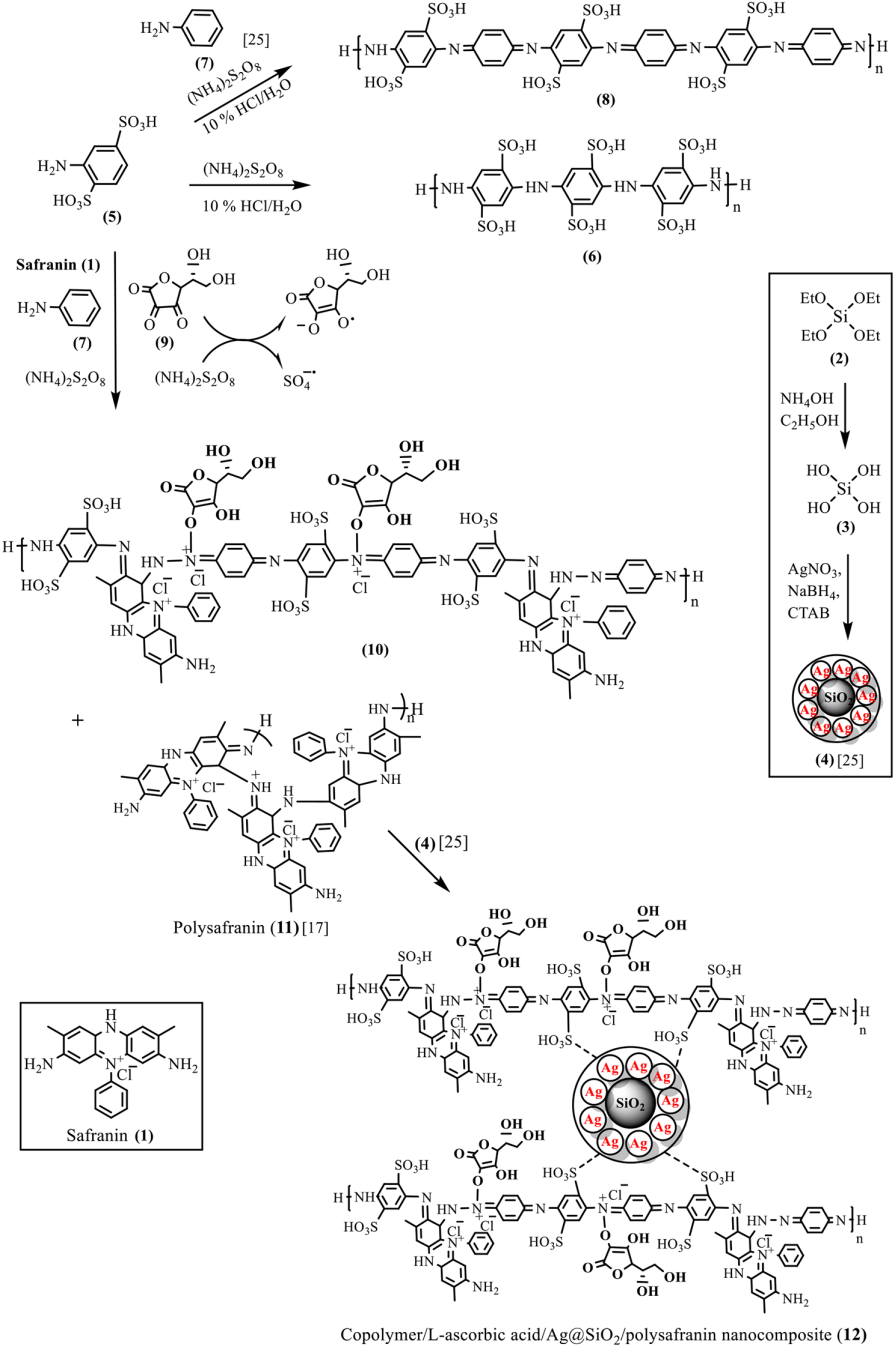
Chemical synthesis of copolymer/L-ascorbic acid/Ag@SiO_2_/polysafranin nanocomposite (**12**)

Organic dyes have similar structural features as conducting polymers except for their low molecular weights, and they are rated as electric insulators [11]. Dyes frequently interact with conducting polymers in four fundamental ways: π–π interactions between aromatic rings, electrostatic cationic/ionic interactions, hydrogen bonding, and hydrophobic interactions [11]. Several trials have reported the influence of the presence of dyes on the morphology and conductivity of polymeric chains or films during preparation. The participation of dyes during polymer preparation significantly affects the resulting conductivity negatively or positively, as exemplified in polypyrrole and its composites [12, 13]. In the presence of a low concentration of methyl orange dye, the conductivity of polypyrrole increased, but the reverse was true when the dye concentration increased [14]. Convincing support for better morphology and conductivity enhancement of the polymer in the presence of dyes has not yet been provided in the literature; however, it is assumed that dyes assist in intermolecular charge transport in chains via their conjugated molecular structure. It is worth noting that dyes have a limited influence on the conductivity of polyaniline [15, 16]. Safranin, 3,7-diamino-2,8-dimethyl-5-phenylphenazinium chloride (**1**), as shown in Fig. 1, is a well-known cationic dye with various applications [17]. Chemical oxidation of dyes containing primary amino group(s) on the benzenoid ring, as occurs for safranin, is expected to produce a conducting polymer or composite from the dye [18]; however, the oxidation of safranin itself leads to a nonconducting oligomer [19]. Notably, the preparation of copolyaniline using aniline and a dye as monomers has led to products with reduced conductivity and unresolved morphology [20].

As part of an ongoing project directed to research the use of PANI and its functionalized analogues as multifunctional substrates for industrial application [21–24], we recently reported the chemical synthesis and characterization of poly(aniline-co-aniline-2,5-disulfonic acid) and its composite containing L-hexuronic acid (Fig. 1) and metallic Ag/SiO_2_ nanoparticles as an efficient, new, thermally stable anionic polyelectrolyte to remove safranin dye from aqueous media [25]. Uptake rates of up to 82.5% adsorption were achieved within 75 min, and the equilibrium time was 45 min. Additionally, uptake was well defined by the pseudo-second-order model with a rate constant K_2_ = 0.03 g^− 1^ mg^− 1^ min^− 1^ for 19 mg of safranin. A comparison of the safranin adsorption efficiency of the synthesized material with that of other reported materials in the same domain suggested that the composite had a high adsorption rate and capacity.

In the research described in this paper, we investigated the resynthesis of such a highly rich, self-doped polyaniline composite (Fig. 1, compound (**12**)) in the presence of safranin by oxidative polymerization and investigated the influence of the incorporation of the dye on achieving better conductivity. Slightly different from our reported synthesis of its analogue [25] and to ensure a better silver-doped silicacomplex sphere morphology, cetyltrimethylammonium ammonium bromide (CTAB) was used as an emulsifier. Modification of the polymer properties by incorporating safranin dye, we proposed, would help to achieve better conductivity. To our knowledge, no studies have investigated the influence of incorporating safranin dye with sulfonated PANI or polyaniline derivatives/Ag nanocomposites to achieve better conductivity.

Interestingly, according to the literature data, the oxidation of safranin itself or its copolymerized material usually impairs the material’s conductivity; however, our results described here indicated that incorporating the dye with the highly sulfonated polyaniline derivatives/Ag@SO_2_ nanocomposite (Fig. 1, compound (**12**)) resulted in better conductivity after heating. The examined material changed from a semiconductor to a metallic material, and its DC conductivities were comparable to those of many commercial inorganic or organic composites. To understand the conduction mechanism, we also studied the electrical conductivity vs. 1000 / T (K) at various frequencies, the electric modulus characterization and the permittivity, ε′, vs. frequency.

## Experimental

### Materials

Commercial aniline (AlGomhoria Chemicals Co.; Egypt), 2-aminobenzene-1,4-disulfonic acid (ICI; Manchester, UK), safranin (≥ 85%; Merck, Darmstadt, Germany), ammonium persulfate (Oxford Lab Fine Chemicals, India), and tetraethyl orthosilicate (TEOS) (98%; Sigma‒Aldrich, Steinheim, Germany) were used. Cetyltrimethylammonium ammonium bromide (CTAB 98%, Sigma‒Aldrich, Steinheim, Germany) was used. Silver nitrate (99.9%, HOLPRO ANALYTICS DIVISION, Midrand, INDIA) and sodium borohydride (95%, Fluka, Switzerland) were used. The ethanol and ammonia solutions (30%) used were of analytical grade. All the chemicals were used without further purification [25]. .

### Measurements

Infrared spectra (IR, KBr pellets; 3 mm thickness) were recorded on a Perkin-Elmer Infrared Spectrophotometer (FTIR 1650). All the spectra were recorded within the wavenumber range of 4000–600 cm^-1^ at 25 °C. Absorption spectra were measured with a UV 500 UV–Vis spectrometer at 16 °C (rt) in DMSO with a polymer concentration of 2 mg/10 mL. Elemental analysis of the as-synthesized copolymer was performed at the Microanalytical Unit, Cairo University. Inherent viscosities (η_inh_) were measured at a concentration of 0.5 g/dL in H_2_SO_4_ at 30 °C by using an Ubbelohde viscometer. Thermogravimetric (TG) and differential thermogravimetric (DTG) analyses were carried out at temperatures ranging from 20 °C to 400 °C in a nitrogen atmosphere by means of a Shimadzu DTG 60 H thermal analyser. The experimental conditions involved a platinum crucible and a nitrogen atmosphere with a 30 mL/min flow rate and a heating rate of 10 C/min. Differential scanning calorimetry (DSC-TGA) analyses were carried out using an SDT-Q600-V20.5-Build-15 instrument at the microanalytical unit of Cairo University. Cyclic voltammetry (CV) was performed using an eDAQ system (www.eDAQ.com, Australia) consisting of an ER466 potentiostat connected to an e-corder that was inputted into eChem software (running on a PC using Microsoft Windows 10). The working electrode was a 3 mm diameter glassy carbon electrode, the reference electrode was Ag/AgCl, and the auxiliary electrode was a 0.25 mm diameter Pt wire. The applied potentials ranged from − 500 to + 500 mV, and the scan rate during one cycle was 100 mV s^-1^. The volume of the voltammetric cell was approximately 15 ml. The polymer powder was pressed to form discs with diameters of 10 mm and thicknesses of 1 mm. Silver electrodes were deposited on both sides of the sample surface by thermal evaporation, and two copper wires were fixed on the sample using conducting silver paint. Energy-dispersive X-ray spectroscopy (EDXS) was used to observe the morphologies of the polymers by scanning electron microscopy (SEM) (JEOL-JSMIT 200, Japan) and transmission electron microscopy (TEM) (JEOL-JTM-1400 plus, Japan) at the E-Microscope Unit, Faculty of Science, Alexandria University. The samples were sonicated in deionized water for 5 min, deposited onto carbon-coated copper mesh and allowed to air dry before examination [21–24].

### Preparation of silver-doped silica complex nanoparticles (4)

The synthesis of silver-doped silica complex spheres (**4**) was slightly modified from a method reported elsewhere [25]. In brief, a mixture of CTAB (1 g), EtOH (25 ml), NH_4_OH (40 mL, 30%), and tetraethyl orthosilicate (TEOS) (**2**)/EtOH (10 ml/20 ml) was magnetically stirred at room temperature for 3 h, after which AgNO_3_ (0.5 g) and NaBH_4_ (250 mg) were added to the in situ-formed tetrahydroxyorthosilicate (**3**), after which the mixture was stirred for 10 h at the same reaction temperature. The crude product (**4**) was collected by centrifugation and worked up as reported previously [25]. The following physical data were recorded: IR (KBr pellets, υ cm^-1^): 3475, 3470, 3467, 2924, 2853, 1739, 1638, 1512, 1480, 1467, 1427, 1420, 1405, 1398, 1229, 1083, 964, 798, 455, 729, 720, 711, 697, 689, 667, 558, and 537. UV‒Vis (λ_max_ nm): 410 nm.

### In situ preparation of poly(aniline-co-aniline-2,5-disulfonic acid)/L-ascorbic acid/Ag@SiO_2_/polysafranin nanocomposite (12)

**Fig. 2:**
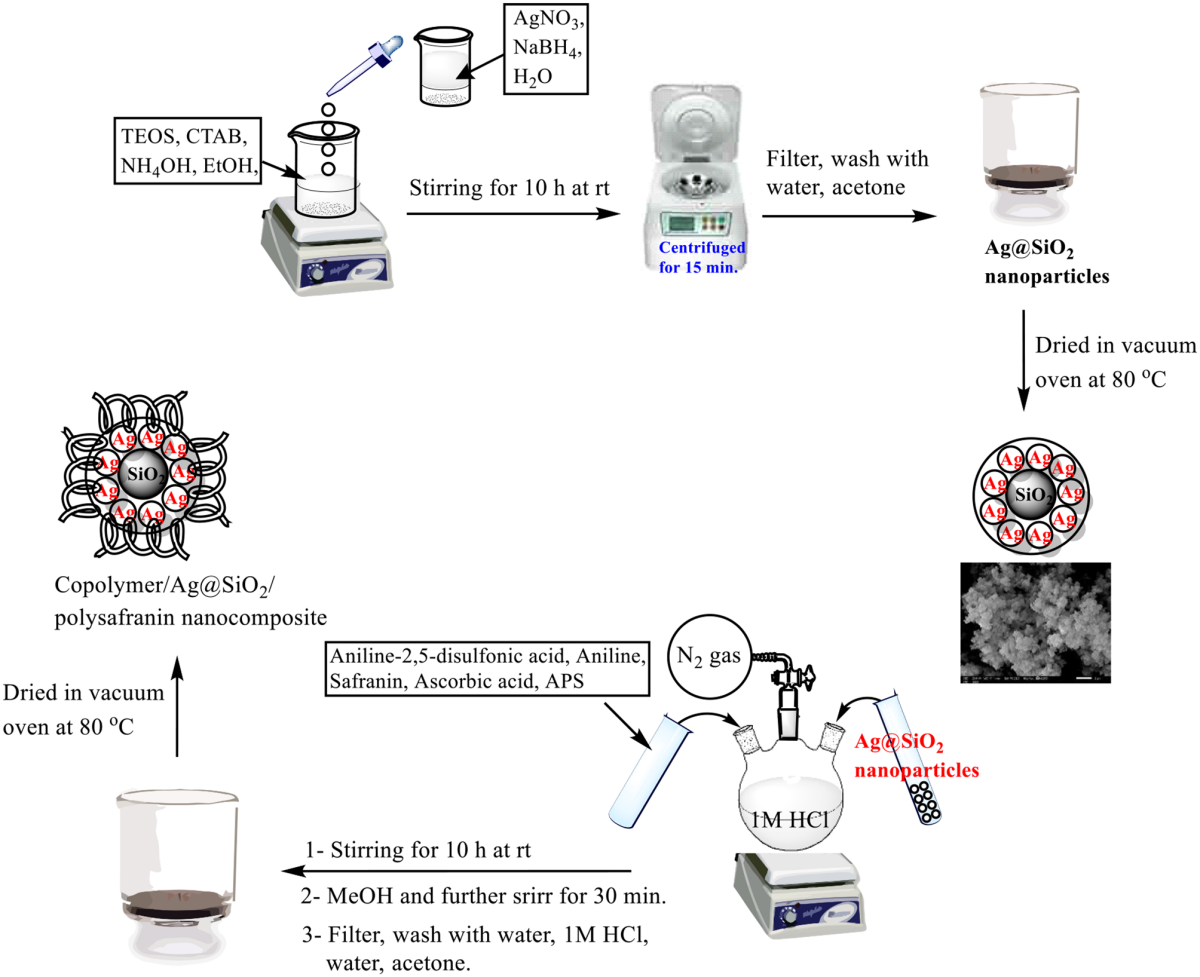
In situ preparation of poly(aniline-co-aniline-2,5-disulfonic acid)/L-ascorbic acid/Ag@SiO_2_/polysafranin nanocomposite (**12**)

The preparation of the targeted nanocomposite (**12**) has been reported elsewhere [25]. The synthesis of (**12**) in this work was performed in the presence of safranin dye (**1**). In brief, aniline-2,5-disulfonic acid (**5**) (12.65 g, 0.05 mol), aniline (**7)** (2.00 g, 0.0215 mol), safranin (**1**) (1 g, 0.0028 mol), and ascorbic acid (**9**) (2.00 g, 0.011 mol) were added to an aqueous 10% HCl (500 ml) solution, followed by the slow addition of ammonium persulfate (15.0 g, 0.0657 mol). A colour change pattern was clearly observed during the polymerization of the copolymer mixture (**10**). Without isolation, silver-doped silica (**4**) (0.5 g) was added to the mixture, and stirring was continued for an additional 10 h. Polymerization was stopped by the addition of CH_3_OH (50 ml), and the precipitate (**12**) was generated as reported previously [25]. The process of in situ preparation of poly(aniline-co-aniline-2,5-disulfonic acid)/L-ascorbic acid/ Ag@SiO_2_/ polysafranin nanocomposite (**12**) is depicted in Fig. 2. IR (KBr pellets, υ cm^-1^) bands were observed at υ 3479, 3466, 3458, 3437, 3401, 3373, 3305, 3296, 3262, 3255, 3099, 2926, 2855, 1699, 1639, 1607, 1578, 1498, 1410, 1301, 1231, 1155, 1096, 1043, 1015, 881, 817, 802, 758, 704, 665, 631, 598, 587, 577, 568, 560, 539, 506, 465, 459, and 454. Calc. for C_58_H_57_N_12_S_3_O_12_: (1282.3); C, 54.23; H, 4.47; N, 13.08; S, 4.99; Found: C, 54.64; H, 5.58; N, 7.65; S, 5.05.

## Results and discussion

### Preparation of poly(anilinecoaniline-2,5disulfonic acid) (8) [26]

Direct oxidative polymerization of 2,5-aminobenzenedisulfonic acid (**5**) in acidic aqueous media (Fig. 1) led to the formation of the known highly water-soluble oligomer (**6**) [26], which was collected by evaporating the solution to dryness. The inductive and steric effects of sulfonate groups hinder the effective generation of reactive intermediates and lead to early termination of polymer growth. The copolymer (**8**) was chemically prepared from commercial aniline-2,5-disulfonic acid (**5**) and aniline (**7**) (10 wt % of (**5**)) in aqueous HCl media (pH 1.5) using 1.25x equivalent of ammonium persulfate as an oxidizing agent.

### Preparation of silver-doped silica complex nanoparticles (4)

**Fig. 3:**
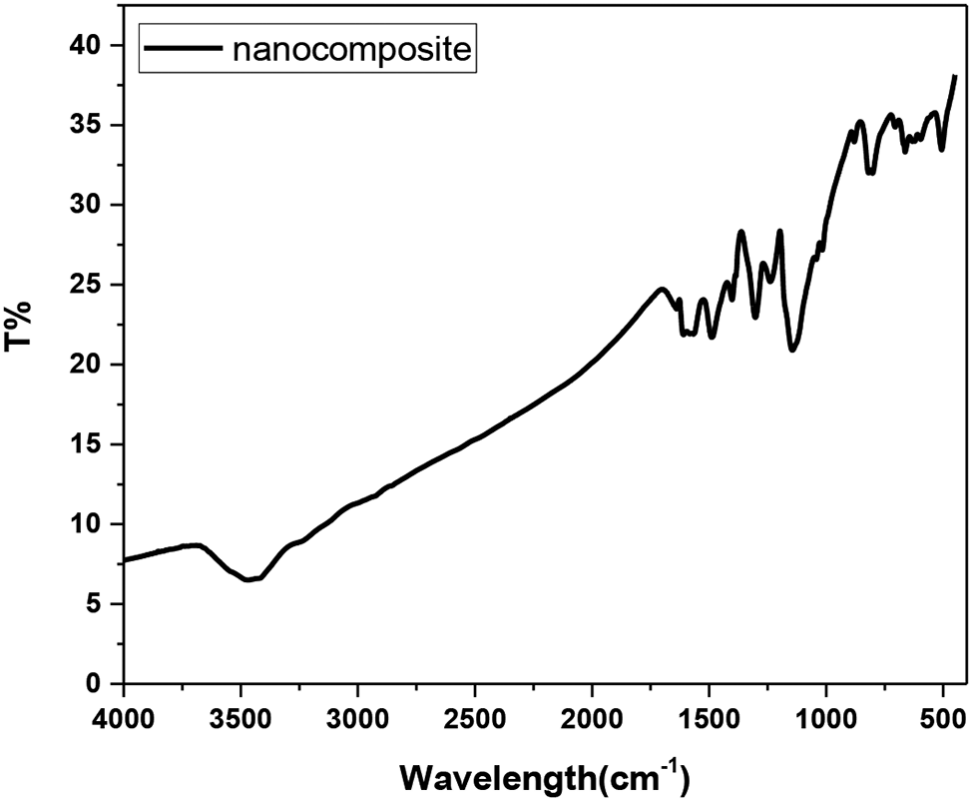
IR spectrum of the Ag@SiO_2_ nanocomposite (**4**)

**Fig. 4:**
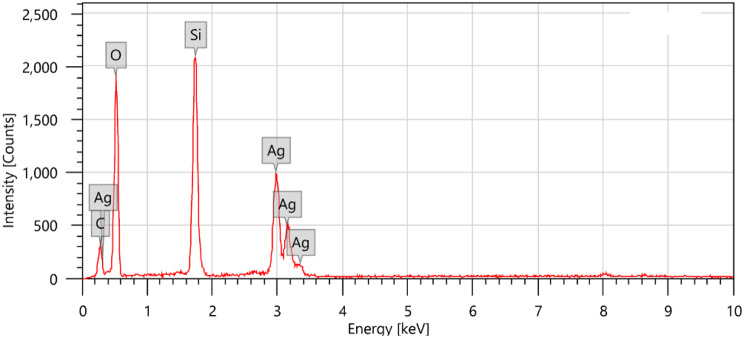
EDXS spectrum of the Ag@SiO_2_ nanocomposite (**4**)

Silver-doped silica complex spheres (**4**) were prepared following a procedure reported elsewhere [25]. Synthesis of (**4**) in this work was performed in the presence of CTAB. Analysis of the IR spectrum (Fig. 3) showed that the CH_2_ stretching vibrational bands of the contaminated CTAB were located at υ2924 and υ2853 cm^-l^. A strong and broad band at υ3470 cm^− 1^ was attributed to the vibrations of the ammonium group in CTAB. The vibrational characteristics of the other functional groups were consistent with the literature [27]. For instance, an observed broad band at υ3467 cm^-1^ was attributed to the vibration of O–H bonds, confirming the presence of Si–OH and/or adsorbed water. The bands at υ1512 cm^-1^ and υ1405 − 1229 cm^-1^ are attributed to NO_3_^–^ ion vibrations. The chemical composition (wt %) of Ag@SiO2 (4) was determined according to the energy-dispersive X-ray spectroscopy (EDXS) spectrum (Fig. 4) to be C, 10.64; O, 39.481; Si, 16.14; and Ag, 33.41, confirming the contamination of the sample with CTAB [25].

**Fig. 5:**
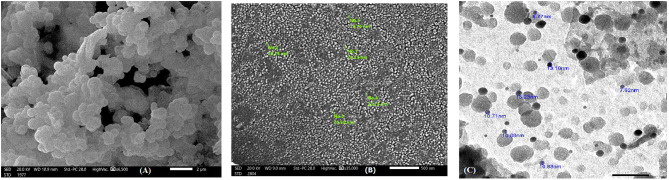
Images of the Ag@ SiO_2_ nanocomposite (**4**): (**a**) SEM image scale bar = 2 nm; (**b**) SEM image scale bar = 500 nm and (**c**) TEM image

Scanning electron microscopy (SEM) and transmission electron microscopy (TEM) images of the SiO_2_@Ag nanocomposite (**4**) are shown in Fig. 5. The particles were nearly spherical in shape and well separated from each other, and the average particle size was 10 nm. Silver particles can be clearly seen embedded in the CTAB-contaminated SiO_2_ matrix in the solid sample image (Fig. 4a and b). According to the TEM image of the prepared Ag@SiO_2_ nanocomposite (Fig. 4c), the SiO_2_ particles were light gray and semispherical with an ordered structure, and the silver nanoparticles (7–13 nm) appeared as dark gray nanoparticles, while the darkness was related to the density of the molecules.

**Fig. 6:**
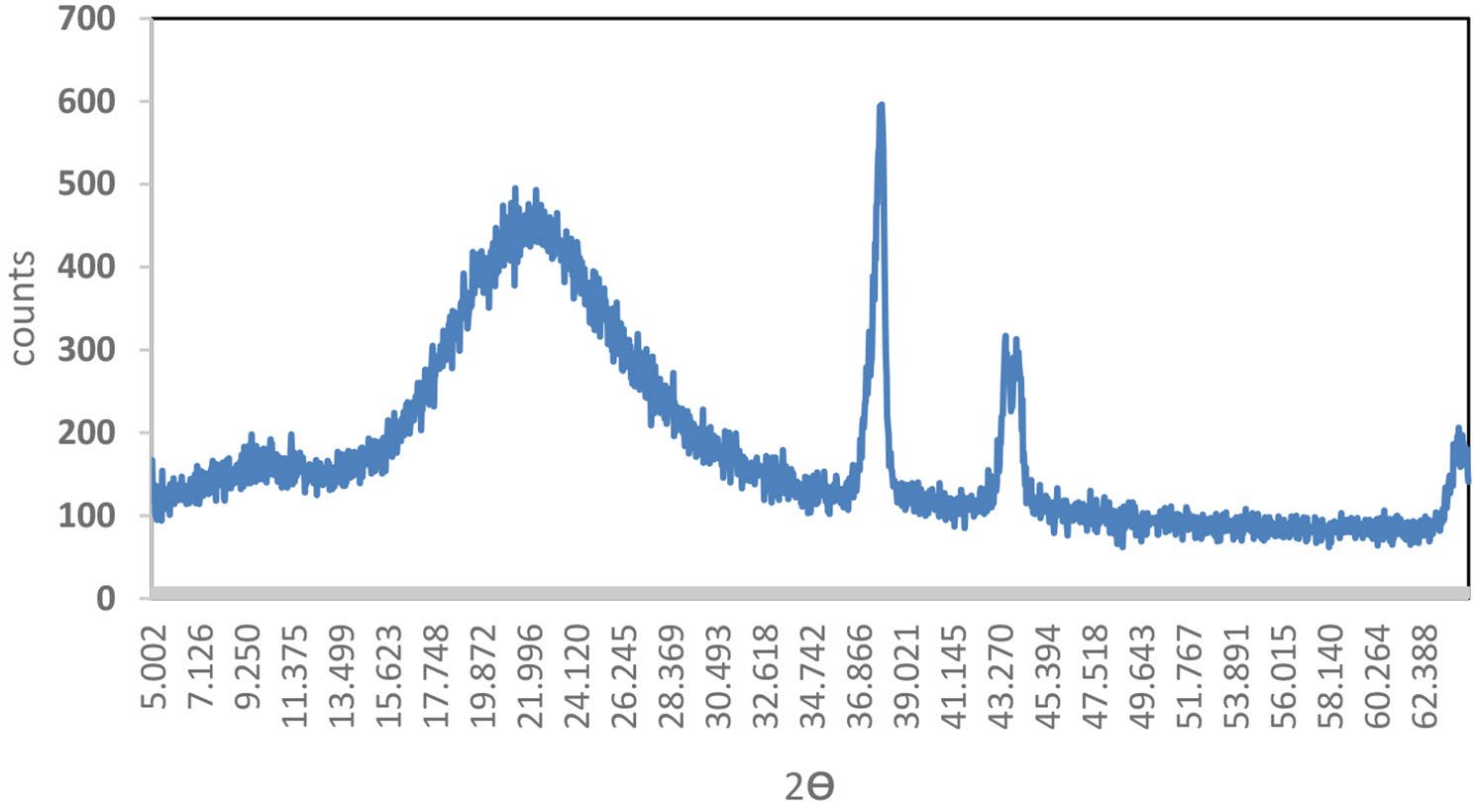
XRD patterns in the 2θ range of 5–80° for the SiO_2_@Ag nanocomposite (**4**)

The XRD spectrum of the Ag@SiO_2_ complex (**4**) (Fig. 6) exhibited characteristic peaks corresponding to silica and silver at 2θ values of 9.49° and 22.3° (SiO_2_ and 37.9° (Ag), 44.1° (Ag), 63.9° (Ag), and 77.2° (Ag), respectively [28]. The calculated interplanar spacing values (d) obtained using Bragg’s law [29] were 0.93 nm, 0.40 nm, 0.24 nm, 0.21 nm, 0.15 nm, and 0.12 nm, while the calculated crystallite (grain) sizes obtained using the Scherrer equation [30] normal to the corresponding planes were 16.66 nm, 16.92 nm, 17.55 nm, 17.91 nm, 19.58 nm, and 21.25 nm, respectively.

### Preparation of the poly(aniline-co-aniline-2,5-disulfonic acid)/L-ascorbic acid/Ag@SiO_2_ polysafranin nanocomposite (12)

**Fig. 7:**
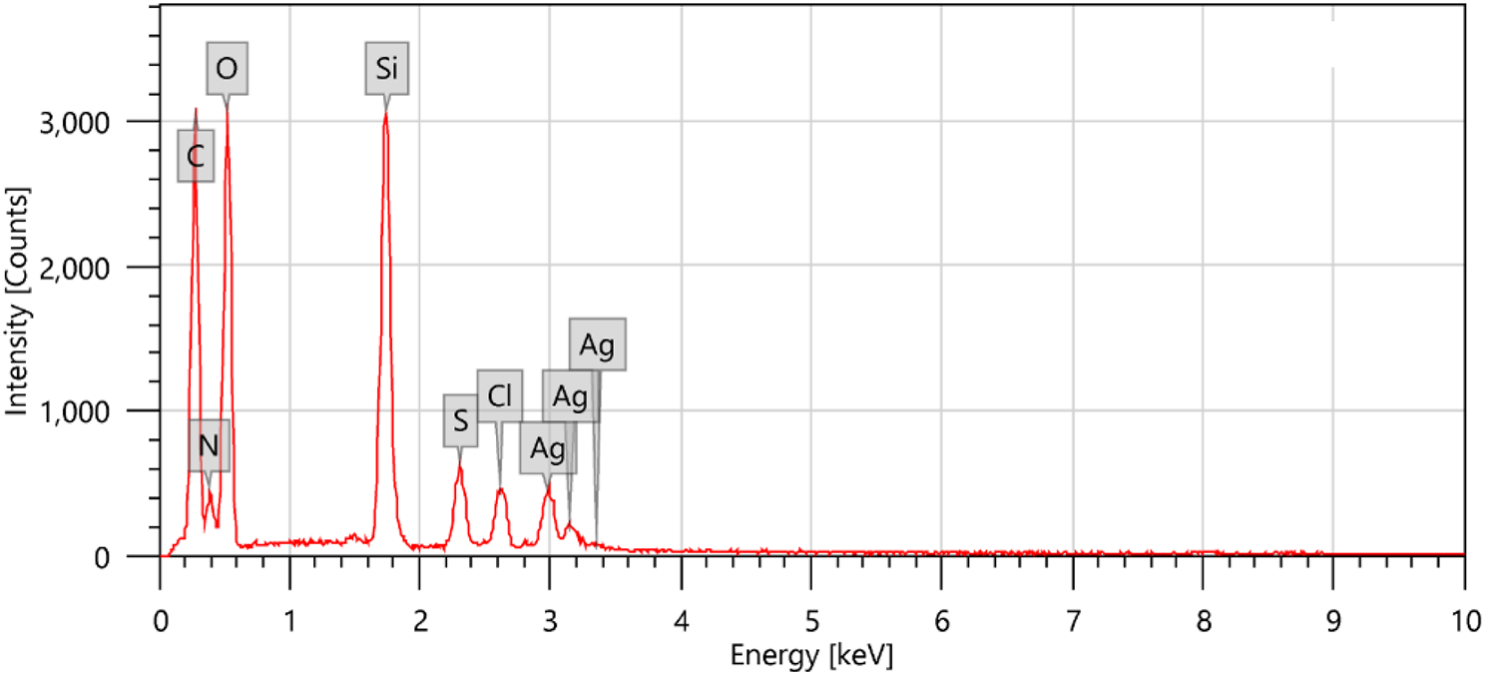
EDXS spectrum of the nanocomposite (**12**)

The copolymer/L-ascorbic acid/Ag@SiO_2_/polysafranin nanocomposite (**12**) was chemically prepared as previously reported [25] in the presence of safranin dye, where the latter was simultaneously oxidized with ammonium peroxydisulfate to actively participate in the copolymer backbone, as proposed, and/or to be converted to its corresponding oligosafranin (**11**) (Fig. 1 [17]), . The calculated elemental composition of the nanocomposite (**12**) was as follows: Calc. for C_58_H_57_N_12_S_2_O_12_: (1282.3); C, 54.23; H, 4.47; N, 13.08; S, 4.99; Found: C, 54.64; H, 5.58; N, 7.65; S, 5.05, confirming the success of the nanocomposite (**12**) preparation. On the other hand, the chemical composition (wt %) of the sample according to the EDXS spectra (Fig. 7) of composite (**12**) was C, 37.86; N, 9.94; S, 1.71; O, 38.95; Si, 6.60; Ag, 3.58; and Cl, 1.36. The EDXS results clearly confirmed that a fraction of the silver particles broke away from the composite surfaces [31].

**Fig. 8:**
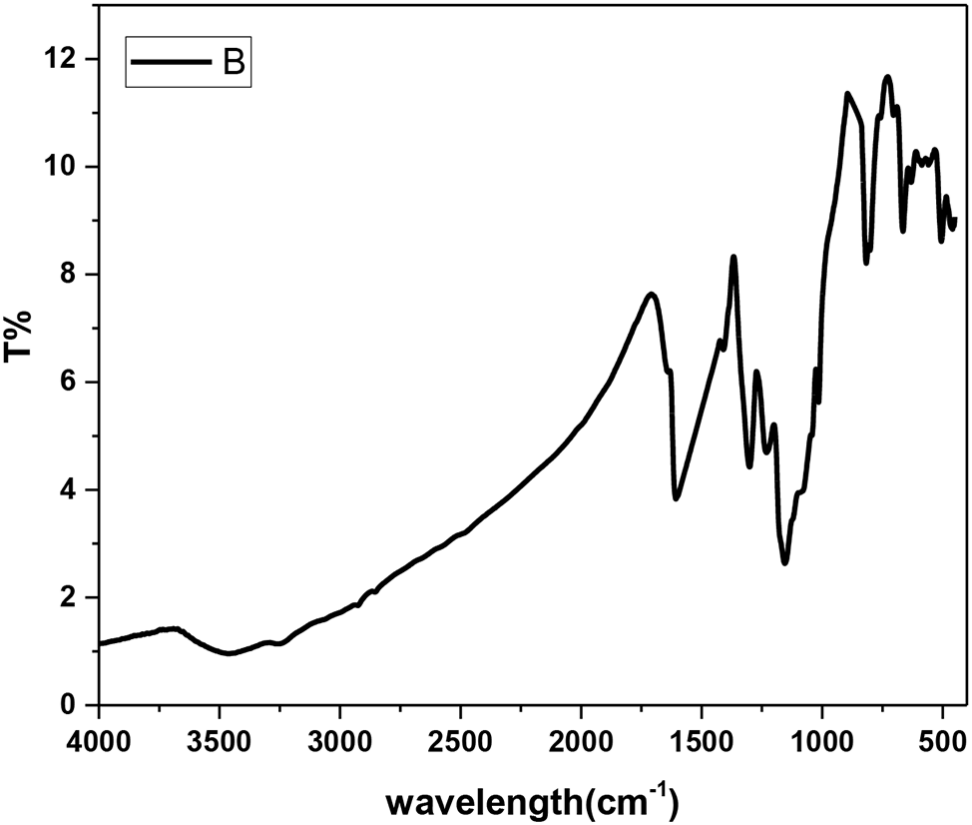
IR spectrum of the nanocomposite (**12**)

**Fig. 9:**
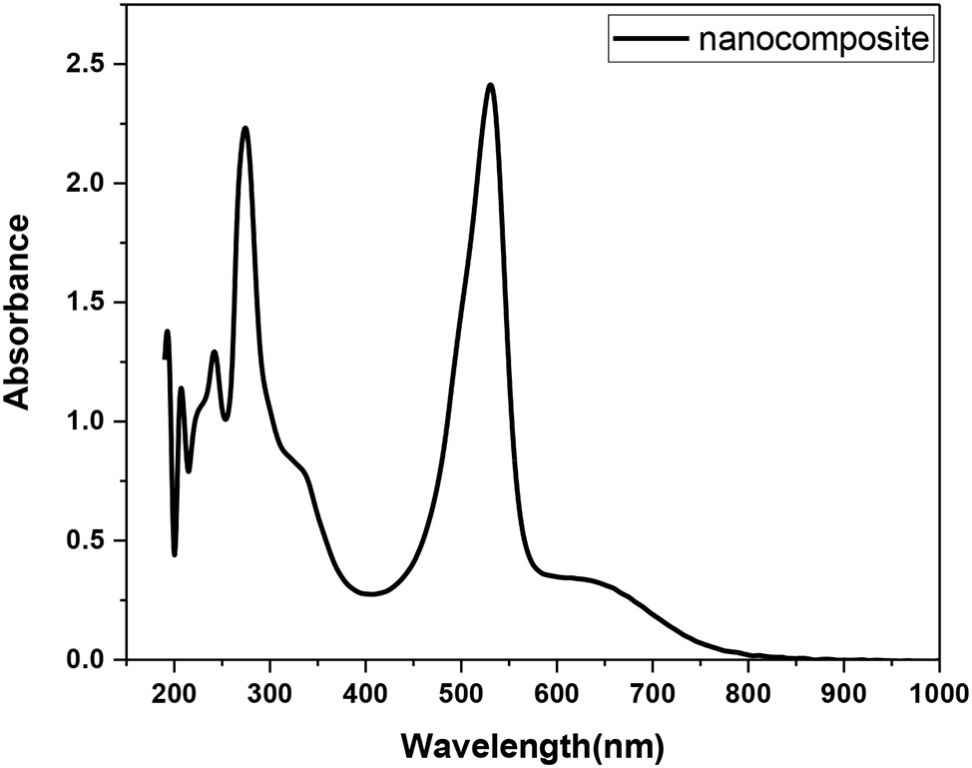
UV-Vis spectrum of the nanocomposite (**12**)

Along with those corresponding to the metallic Ag@SiO2, the spectrum of the nanocomposite **(12)** exhibited IR signals (Fig. 8), vibrational bands, and additional bands assigned to the vibration modes of the NH group, aromatic benzenoid ↔ quinoid structures, and other functional groups such as C-N and O = S = O stretching modes. The vibrational peaks corresponding to ascorbic acid appeared at υ 3479 (–OH), υ 1699 (C–O stretching), υ 1410 and υ 1231 (C–O–C stretching), υ 707 and υ 598 (–OH out-of-plane deformation). Peaks corresponding to the safranin dye appeared at υ 3466 (N-H)_asym_, υ 3373 (N-H)_sym_, 3305 (N-H…H-bonded), υ 3099 (C = C), υ 1639 (NH_2_ scissoring), υ 1607 (C = C), υ 1498 (C = C), υ 1410 (ring aromatic stretching), and υ 802 (aromatic deformation). The UV‒vis spectrum of the nanocomposite (**12**) (Fig. 9) displayed absorption bands at λ 275 nm (π–π* transition), λ 355 nm, λ530 nm (π–π* transition) [32], and λ 655 nm, and their bandgap energies were 4.51 eV, 3.70 eV, 2.34 eV, and 1.89 eV, respectively. The bandgap energies were calculated from the equation ΔE = hc/λ,, where ΔE is the bandgap energy (eV), h = 6.625 × 10^-34^ JS, c = 3 × 10^8^ m/s, and λ is the wavelength. Notably, the absorption spectrum of safranin showed its characteristic peak at λ 520 nm due to the n-π* transition [33]; thus, the observed slightly redshifted band at λ 530 nm in the nanocomposite (**7**) suggested an interaction with safranin.

**Fig. 10:**
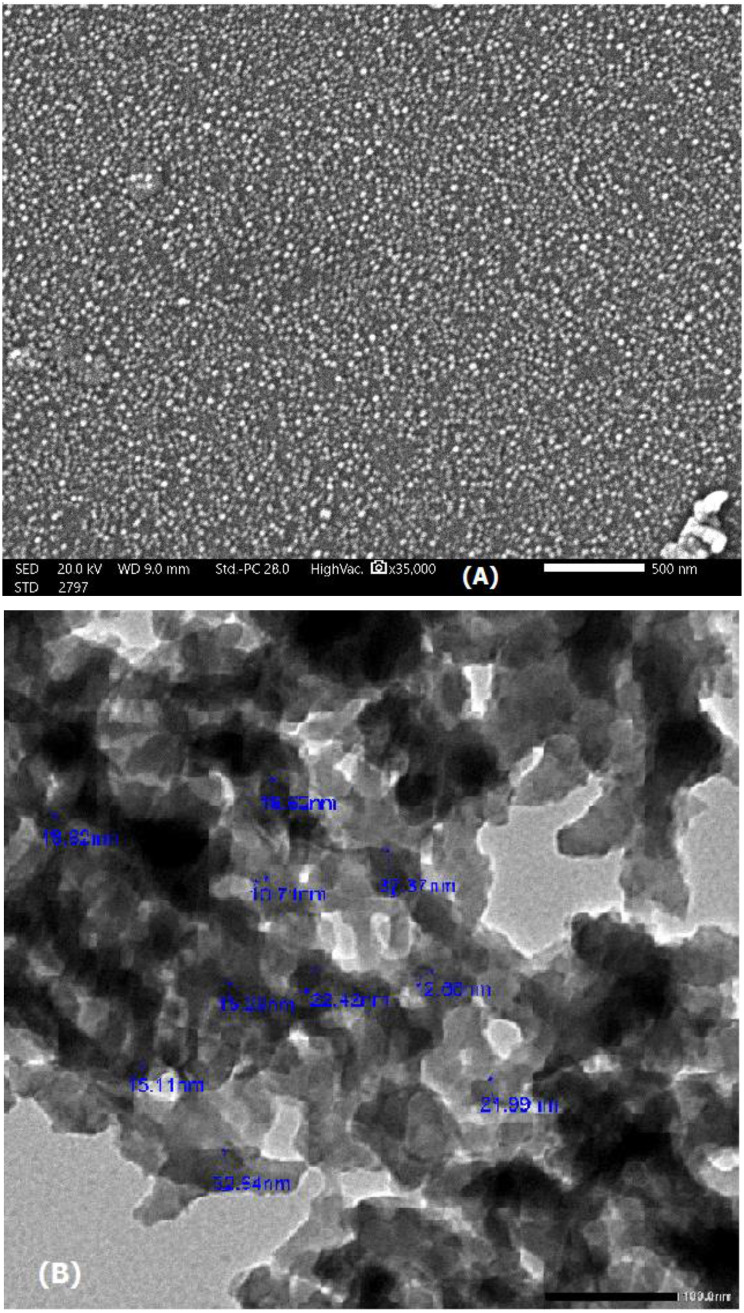
(**A**) SEM and (**B**) TEM images of the nanocomposite (**12**)

SEM images of the copolymer/SiO_2_@Ag nanocomposite **(12)** are shown in Fig. 10. As shown in Fig. 9 (A), the sample microstructure included particles that were almost spherical in shape and had a uniform size, with an average of ∼20 nm. TEM analysis (Fig. 9 (B)) revealed an aggregate of mixed light/dark gray semispherical particles with an average diameter of 20 nm due to CTAB contamination.

**Fig. 11:**
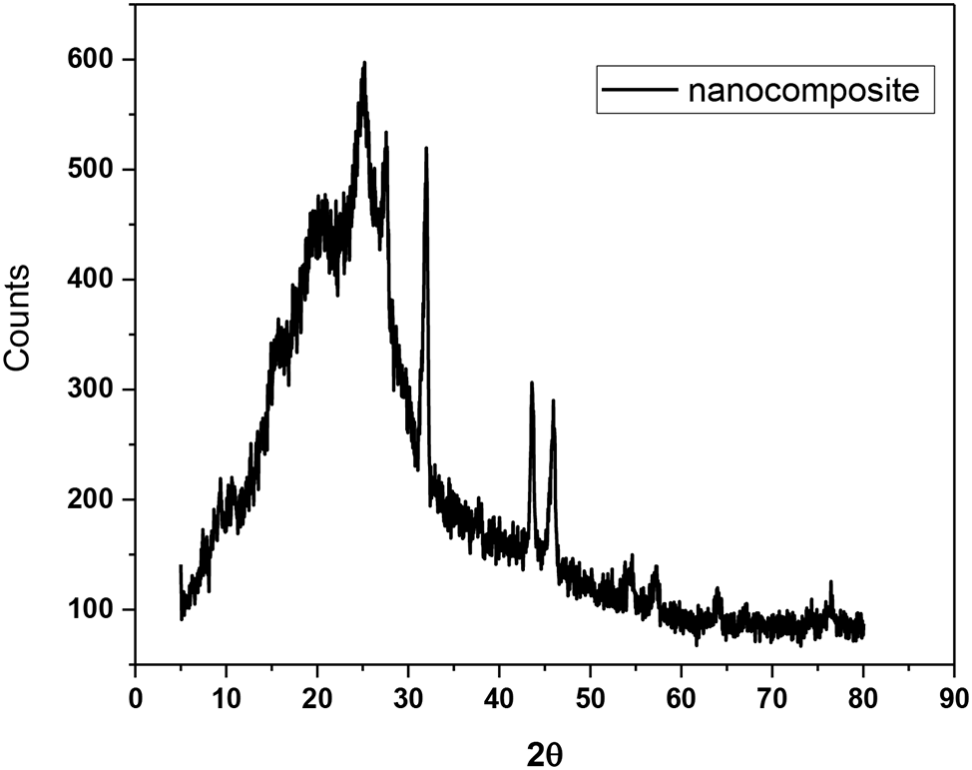
XRD pattern of the nanocomposite (**12**) in the 2θ range of 5–80°

In addition to the main amorphous organic copolymer content, the XRD spectrum of the nanocomposite (**12**) **(**Fig. 11) exhibited characteristic peaks of amorphous silica and crystalline silver at 2q values of 9.34°, 10.67°, 13.43°, 14.56°, 15.25°, 15.71°, 17.29°, 18.81°, 22.90° (SiO_2_), 25.21°, 27.58°, 31.98° (Ag), 43.57°, 45.94° (Ag), 54.61°, 57.32°, 76.43° (Ag), and 77.86° (Ag).

A broad peak observed between 9.34° and 18.81° 2θ degrees is associated with the amorphous nanocomposite (**12**). Interestingly, the characteristic 2θ values due to van der Waals distances between the masses of the phenylene rings of the parallel chains in the main organic copolymer in (**12**) were 25.21° and 27.58° [32]. These values corresponding to two of the peaks may be influenced by increased polymer chain separation due to side chain steric hindrance [10]. The calculated interatomic spacing values (d) corresponding to the abovementioned successive peaks are 0.95 nm, 0.83 nm, 0.66 nm, 0.56 nm, 0.51 nm, 0.47 nm, 0.39 nm, 0.35 nm, 0.32 nm, 0.28 nm, 0.21 nm, 0.20 nm, 0.20 nm, 0.17 nm, 0.16 nm, and 0.12 nm, while their calculated crystallite (grain) sizes are 16.66 nm, 16.67 mm, 16.72 nm, 16.74 nm, 16.75 nm, 16.76 nm, 16.79 nm, 16.83 nm, 19.94 nm, 17.01 nm, 17.09 nm, 17.27 nm, 17.88 nm, 18.03 nm, 18.68 nm, 18.92 nm, 21.13, and 21.35 nm, respectively.

**Fig. 12:**
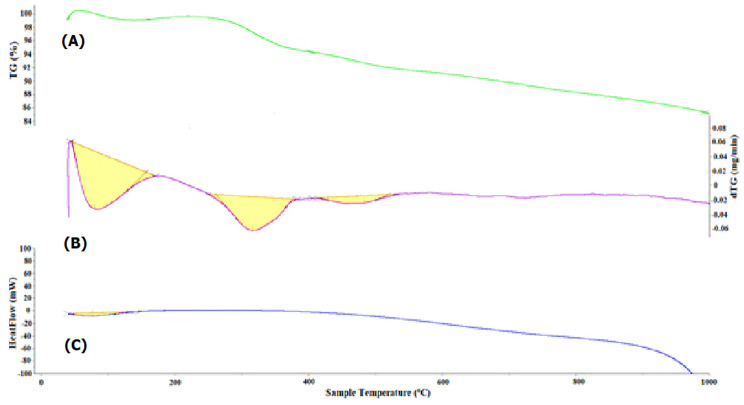
TGA (**A**), DTG (**B**) and DSC (**C**) analyses of the nanocomposite (**12**)

Figure 12 shows the thermogravimetric analysis (TGA), differential thermal analysis (DTA), and differential scanning calorimetry (DSC) curves of the copolymer/L-ascorbic acid/Ag@SiO_2_/polysafranin nanocomposite (**12**). The TGA curve of the nanocomposite (**12**) exhibited successive weight losses at 161 °C (–0.137%), 404 °C (-4.82%) and 530 °C (-8.96%). The DTG spectrum exhibited three endothermic peaks at 170 °C, 383 °C (CTAB removal) [27], and 532 °C, leaving 86.07% of its weight as a residue. The DSC curve shows a weak endothermic peak at 148 °C (energy 127.27 J/g) and a weak broad endothermic peak centred at 380 °C up to 600 °C due to elimination of side chain substituents and subsequent morphological changes. In particular, the DSC spectrum did not display an exothermic degradation peak. This is ascribed to the slow crystallization, and the consequent evolved heat flow over a temperature range was lost in the baseline. This conclusion was also demonstrated by the XRD results for the less ordered and less intense crystalline pattern.

**Fig. 13:**
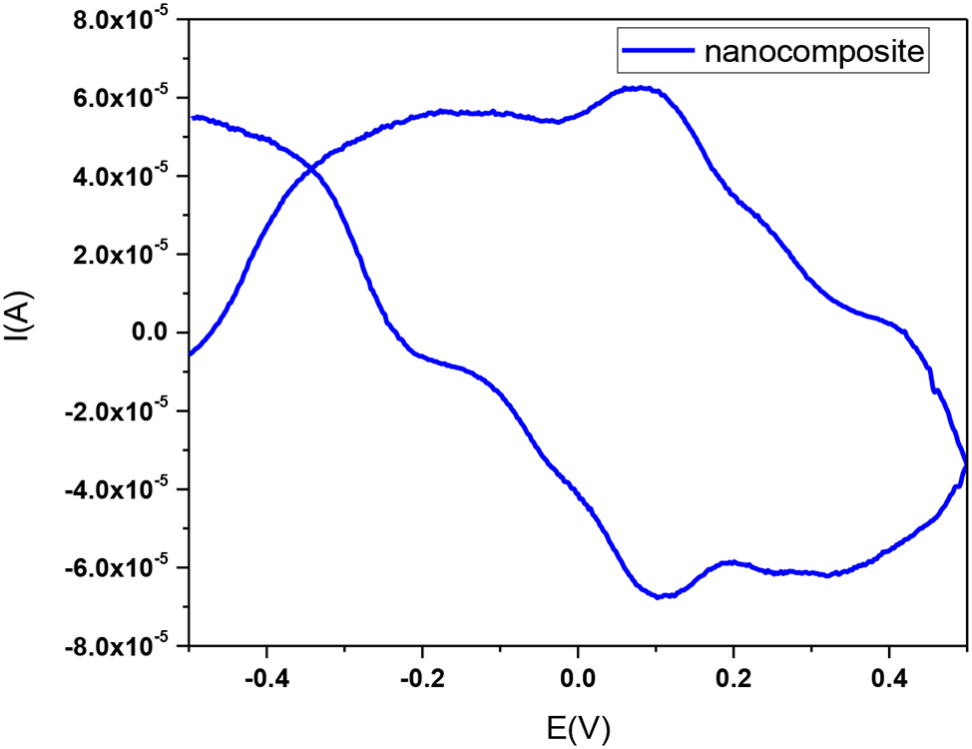
Cyclic voltammogram (50 mV/s) of the nanocomposite (**12**) in DMSO

The cyclic voltammograms (Fig. 13) of the copolymer/L-ascorbic/Ag@SiO_2_/polysafranin nanocomposite (**12**) exhibited four cathodic redox peaks at -5.76 µA/-2.06 V, -0.32.72 µA/-0.04 V, -67.48 µA/-0.11 V, and − 61.23 µA/-0.346 V; correspondingly, four anodic redox peaks were observed at 0.84 µA/0.41 V, 26.06 µA/0.246 V, 61.54 µA/0.10 V, and 55.79 µA/-0.194 V, respectively. The observed redox peaks at 0.11 V and 0.10 V correspond to safranin. The electrochemical behavior of the composite (**12**) was in accordance with that of a reported analog [34].

**Fig. 14:**
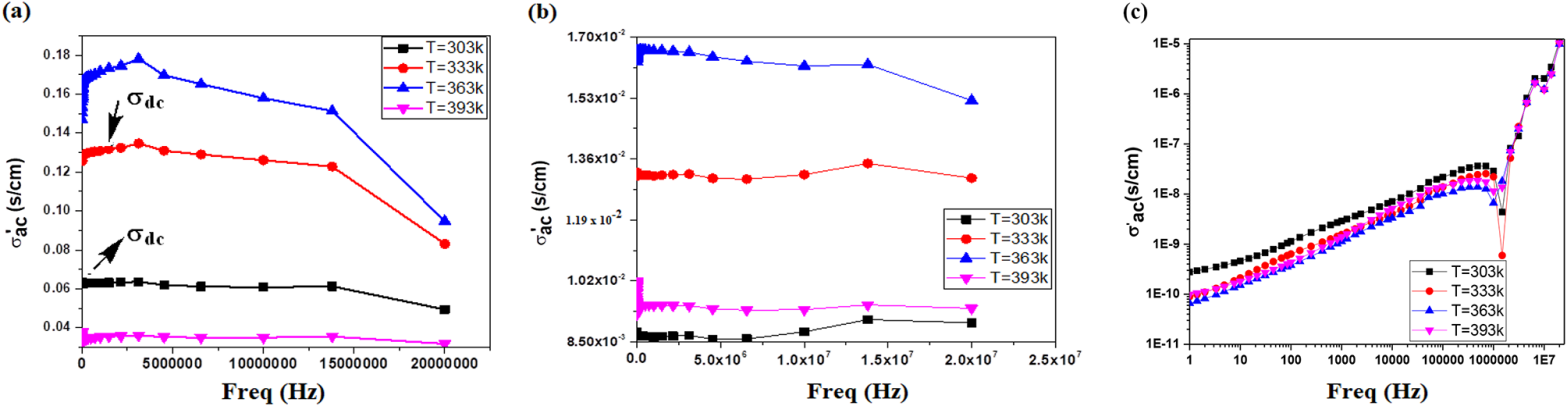
AC electrical conductivity versus frequency (10 to 2 × 10^7^ Hz) in the temperature range (303 K to 393 K) for the (**a**) organic copolymer (**8**), (**b**) copolymer/L-ascorbic/Ag@SiO_2_/ploysafranin nanocomposite (**12**) and (**c**) poly(disulfonated aniline) (**6**)

Electrical conductivity is a critical feature or parameter that provides crucial details on the characteristics of electrical dynamics and transport phenomena in noncrystalline materials. The ac conductivities of our samples at various frequencies (Fig. 14) follow Jonscher’s power law (1) [35]:

1 $${\sigma _{(\omega )}} \, = \, {\sigma _{\rm{dc}}} \, + \, {\rm{A}}{\omega ^{\rm{s}}}$$

where σ_(ω)_ is the total conductivity, σ_(dc)_ is the dc conductivity, ω = 2πf, f is the applied field frequency, s is the constant exponent factor, which takes values between 0< s<1, and A is a temperature-dependent parameter that controls the polarizability strength.

Figure 14 shows the ac electrical conductivity versus frequency from 10 to 2 × 10^**7**^ (Hz) in the 303 K to 393 K temperature range for neat organic poly(aniline-co-aniline-2,5-disulfonic acid (**8**) [25]. Figure 14 (a), poly(aniline-co-aniline-2,5-disulfonic acid)/L-ascorbic acid/Ag@SiO_2_/polysafranin nanocomposite (**12**), Fig. 14 (b) and poly(aniline-2,5-disulfonic acid) (**6**), Fig. 14 (c). The ac conductivity, as shown in Fig. 14, tended to be frequency independent for both the neat polymer (**8**) [25] and its composite (**12**). It is well known that different disordered conductive materials, such as polymers and semiconductors, exhibit similar responses to an applied electric field. Their behaviour is described by the real part, σ′(ω), of the complex electrical conductivity, which is the ac conductivity. At low frequencies, random diffusion of the charge carriers via activated hopping gives rise to a frequency-independent conductivity. The real part of the complex conductivity in the low-frequency regime and in the absence of electrode polarization effects is given by a similar empirical equation, σ′(ω) = σ_dc_[1+ (ω/ω_0_)^n^], where n is the same constant exponent factor. The characteristic frequency, ω_ο_, corresponds to the onset of the ac conductivity and is a characteristic hopping frequency of those ions contributing to the conductivity. At frequency ω_ο_; σ′(ω_0_) = σ_d_. The total conductivity σ_(ω)_ and the real part of the complex conductivity in the low-frequency regime σ′(ω) are equivalent when σ_dc_ = Aω_o_^n^, where the last relation gives the relationship between dc and ac conductivity. In the intermediate plateau region, the conductivity is almost constant and frequency independent, which is called the dc conductivity σ_dc_ [36]. An increase in temperature increases the mobility (µ) and density (n) of charge carriers, and the dc conductivity is the product of the charge carrier density and mobility according to the following formula: (σ_dc_ = q nµ). This behaviour, we suggest, was because the applied field’s frequency was high enough to synchronize with the hopping frequency. It can be concluded that incorporating safranin dye into highly sulfonated polyaniline derivatives/Ag@SO_2_ nanocomposites achieved better conductivity than did the pure polysafranine analogue [19]. The dc conductivity of the organic oligomer (**8**) [25] varied from 0.06 to 0.016 (s/cm) and that of the composite (**12**) from 0.008 to 0.016 (s/cm) with increasing temperature to 363 K, after which both decreased at 393 K. These findings revealed that when the temperature increased to 363 K, the examined material changed from a semiconductor to a metallic material. As shown in Fig. 10, σ’_ac_ exhibits one anomaly at 363 K corresponding to the phase transitions found by DSC (Fig. 12). This phenomenon could be due to the superposition of many mechanisms, such as the reorientation of the side groups and the increase in the mobility of charge carriers. This phase transition is accompanied by a rapid decrease in σ’_ac_ at > 363 K. It is more than likely that this effect camouflages the expected relaxation process [37]. In particular, the dc conductivities found for the organic oligomer (**8**) and its composite (**12**) were comparable to those of many commercial inorganic or organic composites. For example, the reported dc conductivity of the known manganite compound La_0.62_Eu_0.05_Ba_0.33_Mn_0.85_Fe_0.15_O_3_ was 0.001 (s/cm) [36], while those of the vinyl chloride/vinyl acetate/graphene composite and polystyrene/graphene sheet composite were 0.01 (s/cm) and 1.0 (s/cm), respectively [38].

**Fig. 15:**
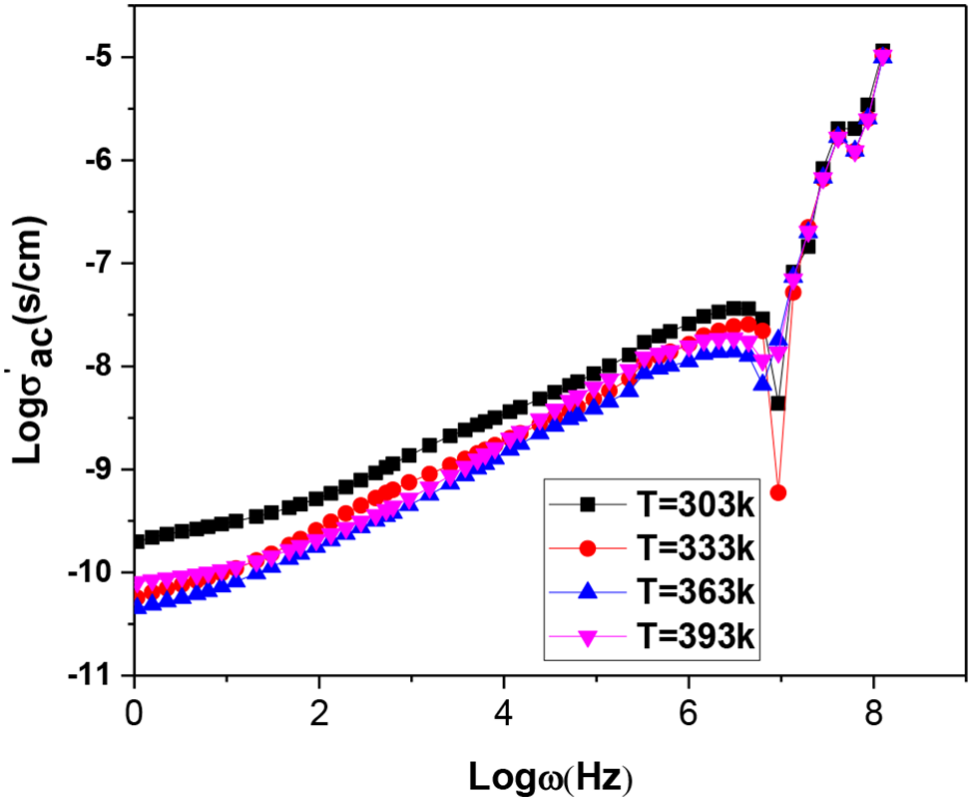
The variation in lnσ_(ω)_ against ln_ω_ for poly(disulfonated aniline) (**6**)

The ac conductivity of the organic oligomer (**6**) increased with increasing frequency, indicating the regular performance of the semiconductor (Fig. 14c). Two distinct trends are observed: at low and high frequencies caused by the electrode in the former case and by the increase in the ionic conductivity in the latter case. The effect of charge carriers travelling over shorter distances increases the ac conductivity as the frequency increases. The exponent factor, s, can be obtained by plotting lnσ_(ω)_ against ln_ω_, as shown in Fig. 15, where s is the slope of the obtained line. The dc conductivity, A parameter and the values of s power in the temperature range 303 K to 393 K were σ’_ac_ 1.2 × 10^− 7^ (s/cm), A; -9.89, s; 0.36 at 303 K; σ’_ac_ 1.2 × 10^− 7^ (s/cm), -10.38, s; 0.42 at 333 K; σ’_ac_ 1.2 × 10^− 7^ (s/cm), -10.53, s; 0.425 at 363 K; and σ’_ac_ 1.2 × 10^− 7^ (s/cm), -10.39, s; 0.426 at 393 K, respectively. It is observed that S tends to increase with increasing temperature. This behaviour is due to nonoverlapping small polaron tunnelling [36, 37]. This variation as a function of temperature is consistent with a thermally triggered process.

**Fig. 16:**
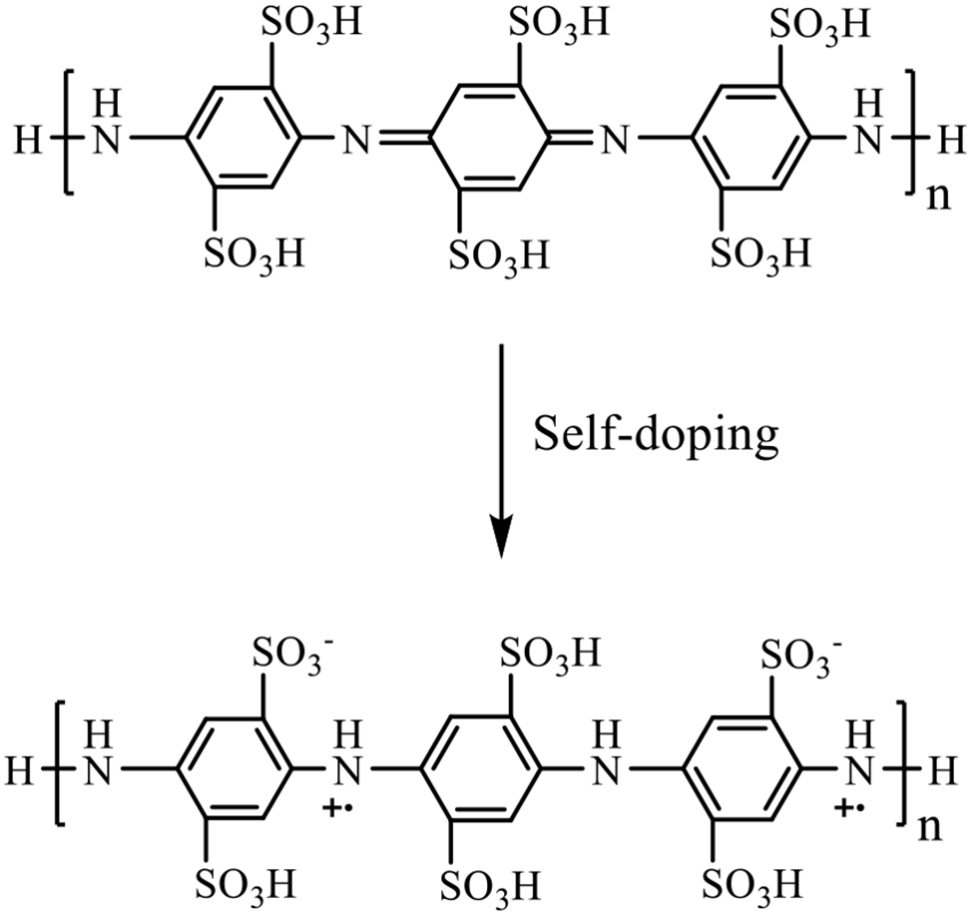
Proposed self-doping effect of the poly (disulfonated aniline) (**6**)

The observed ac conductivity of the organic oligomer (**6**) (Fig. 14c) can be attributed mainly to the effect of self-doping between the sulfonate side groups and the positively charged nitrogen atoms in the polymer chains [34], as depicted in Fig. 16.

**Fig. 17:**
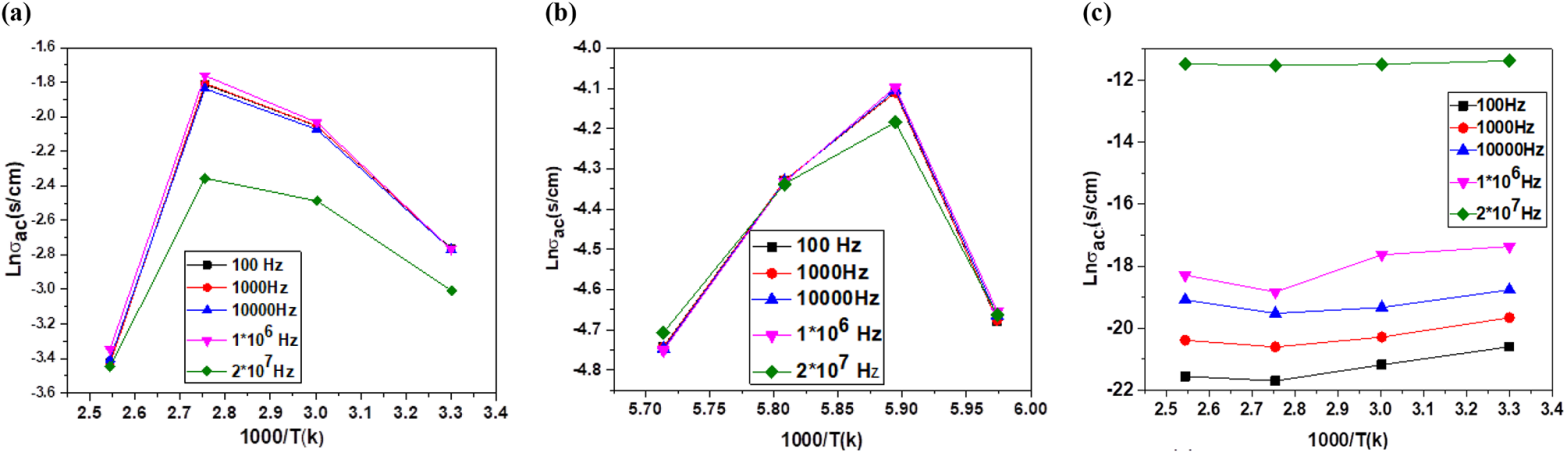
Plots of Ln_ϭac_ vs. 1000/T (K) at various frequencies for the (**a**) organic copolymer (**8**), (**b**) copolymer/L-ascorbic acid/Ag@SiO_2_/polysafranin nanocomposite (**12**) and (**c**) poly(disulfonated aniline) (**6**)

The dependence of the electrical conductivity on the grain and grain boundaries of the oligomers (**6**), (**8**) and the composite (**12**) in the 303–393 K temperature range and their temperature dependence could be fitted to the usual Arrhenius Eq. (2) (Fig. 17).

2 $$\sigma ={\sigma }_{o}exp\left({-E}_{a\sigma }/KT\right)$$

where E_aϭ_ is the activation energy.

The Arrhenius equation can be written in a nonexponential form and is often more convenient to use and interpret graphically; see Eq. (3).

3 $${\rm{ln}}{\sigma _{{\rm{ac}}}}\, = \,{\rm{ln}}{\sigma _0}\, - \,{{\rm{E}}_{\rm{a}}}\sigma /{\rm{KT}}$$

Figure 17 shows the graphs of Ln_ϭac_ versus 1000/T (K) at various frequencies for organic poly(aniline-co-aniline-2,5-disulfonic acid (**8**) [25] (Fig. 17 (a)), the copolymer/L-ascorbic acid/Ag@SiO_2_/polysafranin nanocomposite (**12**) (Fig. 17 (b)) and poly(aniline-2,5-disulfonic acid) (Fig. 17 (c)). The electrical conductivity tended to increase with increasing temperature within the 303–393 K range, indicating semiconducting behaviour for all the compositions studied. As shown in the figure, there were two distinct regions with two different slopes. The thermal activation energies were calculated from the slopes of the two regions. The values of the activation energy E_a1_ from 303 to 333 K and E_a2_ from 333 to 393 K are tabulated in Table 1. The two distinct conduction regions correspond to two different conduction mechanisms: one can be attributed to limited grain boundary scattering, and the second is referred to as variable range hopping. Nearly all the carriers are inhibited by grain boundary effects at low temperatures. The thermal energy of the charge carriers increases with temperature, facilitating them to easily hop over obstacles caused by imperfections accumulating at grain boundaries.

**Table 1:** Values of activation energy for the organic copolymer (**8**) [25] and copolymer/L-ascorbic acid/Ag@SiO_2_/polysafranin nanocomposite (**12**)

Polymer	E_a1_^i^ (eV)	E_a2_^ii^ (eV)
Oligomer (**6**)	2.58	2.63
Oligomer (**8**)	7.53	1.2
Composite (**12**)	2.277	0.967

^i^ E_a1_ from (303–333 K)

^ii^ E_a2_ from (333–393 K)

Generally, the relation [σ’_ac_ (ω,T) = Aω^s(ω,T)^] represents the electrical conductivity described by the Jonscher power law model, and s(T) represents the power exponent, which depends on the temperature satisfying the condition 0 ≤ s(T) ≤ 1. In the case of polymers, relaxation process analysis through this formalism is not applicable, especially at low frequencies and high temperatures, because the dc conductivity phenomenon hides any relaxation process [37]. To avoid such obstacles, the electric modulus formalism was defined as the inverse of the relative complex permittivity ε*(ω,T), as calculated as given in Eq. (4) [39].

4 $$M\ast\, = \,1/\varepsilon\ast \,(\omega ,{\rm{T}})\, = \,{M^{\prime}}(\omega ,{\rm{T}})\, + \,{jM^{\prime \prime}}(\omega ,{\rm{T}})$$

where *M’* and *M’’* are the real and imaginary parts of the complex modulus and are defined by Eqs. (5) and (6), respectively [37].

5 $${M^{\prime}}\, = \,{\varepsilon ^{\prime}}\,(\omega ,\,{\rm{T}})\,/\,{\varepsilon ^{\prime 2}}\,(\omega ,\,{\rm{T}})\, + \,{\varepsilon ^{\prime \prime 2}}\,(\omega ,\,{\rm{T}})$$

6 $${M^{\prime \prime}}\, = \, - {\varepsilon ^{\prime \prime}}\,(\omega ,\,{\rm{T}})\,/\,{\varepsilon ^{\prime 2}}(\omega ,\,{\rm{T}})\, + \,{\varepsilon ^{\prime \prime 2}}(\omega ,\,{\rm{T}})$$

ε’ (ω, T) and ε’‘(ω, T) are the real and imaginary parts of the complex permittivity, which represent the storage and the subsequent losses of energy, respectively, during every electric field cycle.

To study the electrical relaxation behaviour of conducting materials, electrical modulus characterization is commonly used. The complex electric modulus is measured by the inverse of the complex permittivity [37, 40]. The electric modulus analysis eliminates unwanted capacitance effects caused by electrode contacts and provides a good picture of dc conduction and dipole relaxation. Figure 17 shows the plots of the real M^’^ of the electric modulus versus frequency for the organic poly(aniline-co-aniline-2,5-disulfonic acid (**8**) [25] composite (Fig. 17 (a)), copolymer/L-ascorbic acid/Ag@SiO_2_/polysafranin nanocomposite (**12**) (Fig. 17 (b)) and poly(aniline-2,5-disulfonic acid) (**6**) (Fig. 17 (c)). As the frequency increased for the various temperatures, the real part of the electric modulus exhibited an increase in value but decreased for increasing T up to 363 K and then increased. The anomaly M’ at 363 K corresponded to the phase transitions found by the DSC (Fig. 12). This phenomenon could be attributed to the reorientation of the side groups and the increase in the mobility of charge carriers. The phase transition is accompanied by a rapid decrease in M’ at temperatures > 363 K. Furthermore, for each temperature, M^’^ reaches its highest value at high frequencies, indicating a relaxation process [37, 41]. The charge carriers were movable over great distances at frequencies below the maximum limit. This may correspond to a conduction phenomenon caused by the short-range mobility of charge carriers [38].

The complex dielectric function, ε^∗^(ν), and the complex conductivity function, σ^∗^(ν), were both evaluated via broadband dielectric spectroscopy (BDS). According to Eqs. (7) and (8), these factors are linked to each other [42].

7 $${\varepsilon }^{\ast}\left(\omega \right)={\varepsilon }^{{\prime }}\left(\omega \right)-i{{\varepsilon }^{\prime \prime }}\left(\omega \right)$$

8 $${\sigma}^{\ast}\left(\omega \right)=i{\varepsilon }_{0}\omega {\varepsilon }^{\ast}\left(\omega \right)$$

implying that ${\sigma }^{{\prime }}={\varepsilon }_{o}\omega {\varepsilon }^{\prime \prime }$, ${\sigma }^{{\prime }{\prime }}={\varepsilon }_{o}\omega {\varepsilon }^{{\prime }}$, (${{\varepsilon }}_{\text{o}}$is the vacuum permittivity, and ${\omega }(=2{\pi }{\varepsilon })$ is the radial frequency).

**Fig. 18:**
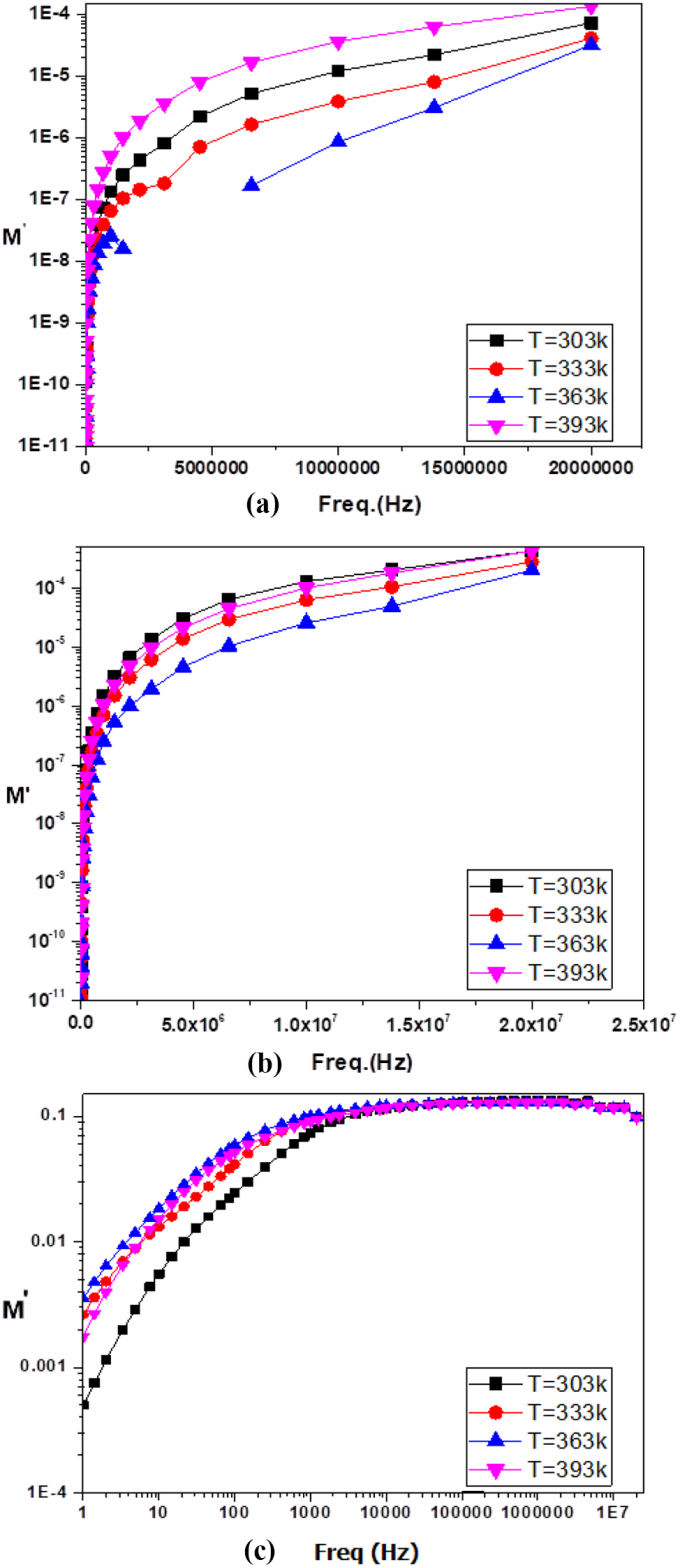
Plots of the real M’ of the electric modulus versus frequency for the (**a**) organic copolymer (**8**), (**b**) copolymer/L-ascorbic acid/Ag@SiO_2_/polysafranin nanocomposite (**12**) and (**c**) poly(disulfonated aniline) (**6**)

**Fig. 19:**
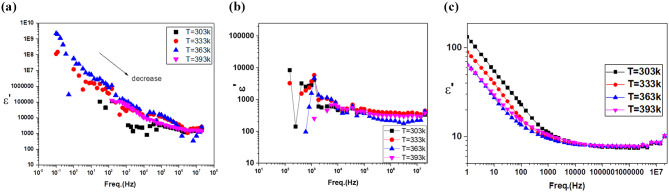
Measured permittivity versus frequency of the (**a**) organic copolymer (**8**), (**b**) copolymer/Ag@SiO_2_/polysafranin nanocomposite (**12**) and

Like with any other kind of spectroscopy, M^’^ spectroscopy involves the use of a fingerprint of the investigated sample. It is distinguished from the other kinds of methods by its wide range of frequencies. This divides the dielectric spectrum into three sectors: molecular dynamics at the microscopic level; charge carrier mobility, which is proven in conductivity mechanisms; and the accumulation of charge carriers at the interfaces at the boundaries between different phases of the multicomponent composites and at the electrode/dielectric material interface. The measured permittivities, ε’, of the prepared samples are graphically plotted against frequency in Fig. 18. The frequency dependence of the permittivity, ε′, showed a marked effect on the frequency window under consideration. However, the figure does not show the marked effect of temperature on the permittivity since all the curves collapse together. The permittivity was essentially independent of the frequency increase for the oligomer (**8**) and composite (**12**). As shown in Fig. 19, ε’ exhibited one anomaly at a temperature of 363 K corresponding to the phase transitions found by DSC (Fig. 12). The variations in ε’ increase with increasing temperature, with a peak at 363 K. The phase transition was accompanied by a rapid decrease in ε’ at temperatures > 363 K. More than likely, this effect camouflaged the expected relaxation process.

The permittivity, ε’, of poly(disulfonated aniline) (**6**) decreased with increasing frequency (Fig. 19c). Two prominent dynamics of the real part of the complex permittivity, ε′ can be seen here as represented against the frequency. In the frequency range (0.1 Hz – 10 kHz), the graph displays a gradual reduction in magnitude. The high permittivity at low frequency is due to electrode polarization. The decrease in ε′ at high frequencies (from 10 kHz up to 20 MHz) was due to the dipoles not having enough time to coincide in motion with the frequency of the applied electric continuous field [42].

## Conclusions

In this work, we report for the first time the synthesis of poly(*aniline-co*-*aniline-2,5-disulfonic acid)*) in a composite containing safranin, L-ascorbic acid, and metallic Ag/SiO_2_ nanoparticles and investigated the influence of incorporating the dye on the morphology and conductivity. The incorporation of L-ascorbic acid had dual effects: as an oxidant and a reactant. Polyanone (*aniline-co-aniline-2,5-disulfonic acid*) was chemically prepared in a low-pH 1.5 aqueous HCl media using 1.25x equivalent of ammonium persulfate oxidant. The silver-doped silica complex spheres were prepared by the first hydrolysis of ethanol-diluted TEOS in the presence of CTAB followed by the subsequent addition of silver nitrate and NaBH_4_. Targeted poly(*aniline-co-aniline-2,5-disulfonic acid*)/polysafranin/L-ascorbic acid/Ag@SiO_2_ nanocomposites were chemically prepared at RT by stirring a mixture of Ag@SiO_2_ nanoparticles, aniline-2,5-disulfonic acid, aniline (10 weight%), safranin, and L-ascorbic acid using 1.25x equivalents of ammonium persulfate oxidant. The elemental composition of the nanocomposite indicated the contribution of (1:3) disulfonated aniline/aniline units to the resulting nanocomposite. The polymeric composite was characterized via IR and UV spectroscopic techniques, cyclic voltammetry, electrical conductivity, and dielectric measurements. SEM, TEM, TGA, and DSC measurements were also performed for additional analysis. The electronic spectrum showed absorption bands attributed to the quinoid ring transition, charge transfer from the HOMO of the benzenoid ring to the LUMO of the quinoid ring and a characteristic safranin absorption peak. EDXS analyses further confirmed not only the contamination of the composite with CTAB traces but also the successful synthesis of the copolymer/SiO_2_@Ag nanocomposite. SEM and TEM images of the SiO_2_@Ag nanospheres revealed well-separated spherical particles with an average size of 10 nm. The surface morphology of the organic copolymer was marked by the presence of hemispherical, well-separated particles with an average size of 21.5 nm. SEM and TEM images of the targeted nanocomposite revealed intensified spherical particles that were dispersed over almost the entire surface, and the SiO_2_/Ag particles were distributed on the composite surface. The DSC curve of these compounds showed a weak endothermic peak at 148 °C (energy 127.27 J/g) and a weak broad endothermic peak centred at 380 °C up to 600 °C due to substituent elimination and subsequent morphological changes. The DSC curve showed a weak endothermic peak at 148 °C (energy 127.27 J/g) and a weak broad endothermic peak centred at 380 °C up to 600 °C due to substituent elimination and subsequent morphological changes. The DSC spectrum did not display an exothermic degradation peak, indicating slow crystallization. The XRD spectrum exhibited peaks corresponding to amorphous silica and crystalline silver at many 2q values, and their interatomic spacing (d) and crystallite (grain) sizes were calculated. The thermal degradation curve of the organic copolymer or the composite exhibited an interesting model of the stability of the polymer, and their subsequent weight loss was completed in four steps, leaving nearly 50% of their weight as a remaining residue. The cyclic voltammogram of the nanocomposite exhibited redox peaks similar to those reported for the aniline/orthanilic acid copolymer. The observed redox peaks at 0.11 V and 0.10 V correspond to safranin.

Because the applied field frequency is high enough to synchronize with the hopping frequency, the ac conductivity tends to be frequency independent for both the copolymer and its composite, where the value of electrical conductivity equals only the d.c conductivity. The d.c. conductivity of the organic oligomer varied from 0.06 − 0.016 (s/cm) and that of the composite from 0.008 to 0.016 (s/cm) with increasing temperature up to 363 K and then decreased at 393 K. The examined material changed from a semiconductor to a metallic material when the temperature was increased up to 363 K. The dc conductivity exhibited one anomaly at a temperature of 363 K corresponding to the phase transitions found by DSC analysis. This phenomenon could be attributed to reorientation of the side groups and an increase in the mobility of the charge carriers. This phase transition was accompanied by a rapid decrease in ac conductivity at this temperature (> 363 K), most likely because of the expected relaxation process. The d.c. conductivities of the examined materials are comparable to those of many commercial inorganic or organic composites. Notably, the conductivity of poly(*aniline-2,5-disulfonic acid)* itself proportionally increased with frequency, indicating the regular performance of the semiconductor. The observed low conductivity is mainly attributed to self-doping between the sulfonate side groups and the positively charged nitrogen atoms in the polymer chains. The electric conductivity/temperature correlation is attributed to two distinct conduction regions corresponding to two different conduction mechanisms, i.e., grain boundary scattering limited and variable range hopping. The frequency dependence of the permittivity, ε′, showed a marked effect on the frequency window under consideration. The permittivity is independent of the frequency increase for the oligomer and the composite. The permittivity of poly(*aniline-2,5-disulfonic acid)* decreased with increasing frequency. This behavior supports the non-Debye dependency by confirming the occurrence of electrode polarization and space charge effects. In conclusion, when dyes participate in the preparation of conducting polymers, they significantly affect both their morphology or conductivity and both. Incorporating safranin dye with thermally stable, highly sulfonated polyaniline derivatives/Ag@SO_2_ nanocomposites achieved better conductivity after heating.

## Data Availability

All data generated or analyzed during this study are included in this published article.

## References

[1] Sebastian J, Samuel JM (2020). Recent advances in the applications of substituted polyanilines and their blends and composites. Polym Bull.

[2] Chapi S (2021). Influence of Co2 + on the structure, Conductivity, and Electrochemical Stability of Poly(Ethylene Oxide)-Based solid polymer Electrolytes: Energy Storage devices. J Elect Mat.

[3] Nasar A, Mashkoor F (2019). Application of polyaniline-based adsorbents for dye removal from water and wastewater – a review. Environ Sci Pollution Res.

[4] Chapi S (2020). Optical, electrical and electrochemical properties of PCL5/ITO transparent conductive films deposited by spin-coating – materials for single-layer devices. J Sci : Adv Mat Devices.

[5] Huang WS, Humphrey BD, MacDiarmid AG (1986). Polyaniline, a novel conducting polymer, morphology and chemistry of its oxidation and reduction in aqueous electrolytes. J Chem Soc Faraday Trans 1 Phys Chem Condens Phases.

[6] Jaymand M (2013). Recent progress in chemical modification of polyaniline. Prog Polym Sci.

[7] Malinauskas A, Self-doped polyanilines (2004). J Pow Sour.

[8] Moulton SE, Pornputtkul Y, Kane-Maguire LA, Wallace GG (2007). Poly(2-methoxyaniline-5-sulfonic Acid)–Surfactant complexes and their Redox and Solvatochromic Behavior. Aust J Chem.

[9] Stilwell DE, Park SM (1988). Electrochemistry of Conductive polymers III. Some physical and Electrochemical Properties Observed from electrochemically grown polyaniline. J Electrochem Soc.

[10] Yue J, Wang ZH, Cromack KR, Epstein AJ, MacDiarmid AG (1991). Effect of Sulfonic Acid Group on Polyaniline Backbone. J Am Chem Soc.

[11] Stejskal J (2020). Interaction of conducting polymers, polyaniline and polypyrrole, with organic dyes: polymer morphology control, dye adsorption and photocatalytic decomposition. Chem Papers.

[12] Nair S, Nair SS, Mishra SK, Kumar DS, Mishra SK, Kumar D. Recent progress in conductive polymeric materials for biomedical applications. Polym Adv Technol. 2019;1–22. 10.1002/pat.4725.

[13] Sapurina I, Li Y, Alekseeva E, Bober P, Trchová M, Morávková Z, Stejskal J (2017). Polypyrrole nanotubes: the tuning of morphology and conductivity. Polymer.

[14] Li Y, Bober P, Trchová M, Stejskal J (2017). Polypyrrole prepared in the presence of methyl orange and ethyl orange: nanotubes versus globules. A comparison study on the improvement of conductivity. J Mater Chem C.

[15] Amer WA, Omran MM, Ayad MM (2019). Acid-free synthesis of polyaniline nanotubes for dual removal of organic dyes from aqueous solutions. Colloid Surf A-Physicochem Eng Asp.

[16] Shi MW, Bai MD, Li BM (2018). Acid Red 27-crosslinked polyaniline with nanofiber structure as electrode material for supercapacitors. Mater Lett.

[17] Ciric-Marjanovi´c G, Blinova NV, Trchova M, Stejskal J (2007). Chemical oxidative polymerization of safranines. J Phys Chem B.

[18] Gouveia-Caridade C, Romeiro A, Brett CMA (2013). Electrochemical and morphological characterization of polyphenazine flms on copper. Appl Surf Sci.

[19] Yang YJ (2016). Facile synthesis of poly(safranine T)/reduced graphene oxide nanocomposite for supercapacitors with wide potential window in aqueous neutral electrolyte. Fuller Nanotube Carbon Nanostruct.

[20] Stejskala J, Prokeš J (2020). Conductivity and morphology of polyaniline and polypyrrole prepared in the presence of organic dyes. Synth Met.

[21] Abd-El-Khalek DE, Hassan HHAM, Ramadan SR (2021). Water-soluble sulfonated polyaniline as multifunctional scaling inhibitor for crystallization control in industrial applications. Chem Eng Res Des.

[22] Zein El-Din AM, Hassan HHAM, Abou El-Kheir MM, Youssef RM (2016). Controlling soil surface crust formation using Nanosized sulfonated polyaniline. J Soil Water Con.

[23] Hassan HHAM, Abd-El-Khalek DE, Abdel Fattah M (2022). Synthesis and assessment of poly (5-nitro-2-aminophenol) as a new scaling inhibitor on controlling the precipitation of CaCO_3_ and CaSO_4_ in solution. J Polym Res.

[24] Hassan HHAM, Abd-El-Khalek DE, Abdel Fattah M (2022). Assessment of self-doped poly (5-nitro-2-orthanilic acid) as a scaling inhibitor to control the precipitation of CaCO_3_ and CaSO_4_ in solution. Sci Rep.

[25] Hassan HHAM, Abdel Fattah M (2023). Efficient removal of safranin from aqueous solution using a new type of metalated highly self-doped polyaniline nanocomposite. Funct Comp Mat.

[26] Tang H, Kitani A, Yamashita T, Ito S (1998). Highly sulfonated polyaniline electrochemically synthesized by polymerizing aniline-2,5-disulfonic acid and copolymerizing it with aniline. Synth Met.

[27] Abduraimova A, Molkenova A, Duisembekova A, Mulikova T, Kanayeva D, Atabaev TS (2021). Cetyltrimethylammonium Bromide (CTAB)-Loaded SiO_2_–Ag Mesoporous Nanocomposite as an efficient Antibacterial Agent. Nanomaterials Nanomaterials.

[28] Sembiring S, Riyanto A, Firdaus I, Junaidi; Situmeang R (2022). Structure and properties of silver-silica composite prepare from rice husk silica and silver nitrate. Ceramics-Silikáty.

[29] Bragg WL (1934). The crystalline state.

[30] Scherrer P (1918). Estimation of the size and Internal Structure of Colloidal Particles by means of Rontgen Rays. Nachr Ges Wiss Göttingen.

[31] Patil K, Wang X, Lin T, Powder, Technol (2013). Electrostatic coating of cashmere guard hair powder to fabrics: silver ion loading and antibacterial properties. Powder Tech.

[32] Karthik R, Meenakshi S (2014). Removal of hexavalent chromium ions using Polyaniline/silica gel composite. J Water Process Eng.

[33] Kumar DA, Shyla JM, Xavier FP. Synthesis and Optical, Photoconductivity Study of Safranin O Dye Sensitized Titania/Silica Oxide System Prepared by Modified Sol-Gel Method, in: 4S ed. Int. J. Recent Technol. Eng. 2018, 7, Blue Eyes Intelligence Engineering & Sciences Publication, pp. 17–22.

[34] Xu Y, Dai L, Chen J, Gal J-Y, Wu H (2007). Synthesis and characterization of aniline and aniline-o-sulfonic acid copolymers. Eur Polym J.

[35] Dhahri A, Dhahri E, Hlil EK. Electrical conductivity and dielectric behavior of nanocrystalline La_0.6_ Gd_0.1_Sr_0.3_Mn_0.75_Si_0.25_O_3_. RSC Adv. 2018;8:9103–11. 10.1039/C8RA00037A. 10.1039/c8ra00037a PMC9078605 35541874

[36] Ncib W, Ben Jazia Kharrat A, Saadi M, Khirouni K, Chniba-Boudjada N, Boujelben W, Structural (2019). AC conductivity, conduction mechanism and dielectric properties of La_0.62_Eu_0.05_Ba_0.33_Mn_0.85_ Fe_0.15_O_3_ ceramic compound. J Mater Sci.

[37] Karoui S, Chouaib H, Kamoun S (2020). Studies of electric, dielectric properties, and conduction mechanism of {(C_2_H_10_N_2_)(MnCl (NCS)_2_)_2n_ polymer. J Phys Org Chem.

[38] Chapi S (2020). Structural and Electrochemical properties of Polymer Blend Based ZnO Nanocomposite Solid Polymer Electrolytes by spin–coating method. J Nano-Electron Phys.

[39] Choudhary S, Sengwa RJ (2018). ZnO nanoparticles dispersed PVA–PVP blend matrix based high performance flexible nanodielectrics for multifunctional microelectronic devices. Curr Appl Phys.

[40] Abutalib MM (2019). Insights into the structural, optical, thermal, dielectric, and electrical properties of PMMA/PANI loaded with graphene oxide nanoparticles. Phys B Condens Matter.

[41] Babaladimath G, Chapi S (2018). Microwave-assisted synthesis, characterization of electrical conducting and electrochemical xanthan gum-graft-polyaniline. J Mat Sci : Mat Electro.

[42] Abd El-Aziz ME, Youssef AM, Abd El-Sayed ES, Moussa MA, Turky GM, Kamel S (2019). Rational design and electrical study of conducting bionanocomposites hydrogel based on chitosan and silver nanoparticles. Int J Bio Macromol.

